# Prognostic analysis of cuproptosis-related gene in triple-negative breast cancer

**DOI:** 10.3389/fimmu.2022.922780

**Published:** 2022-08-01

**Authors:** Shengnan Sha, Luyi Si, Xinrui Wu, Yuanbiao Chen, Hui Xiong, Ying Xu, Wangrui Liu, Haijun Mei, Tao Wang, Mei Li

**Affiliations:** ^1^ Department of Oncology, Affiliated Hospital of Nantong University, Medical School of Nantong University, Nantong, China; ^2^ Department of General Surgery, Affiliated Hospital of Nantong University, Medical School of Nantong University, Nantong, China; ^3^ Department of Clinical Medicine, Medical School of Nantong University, Nantong, China; ^4^ Department of Neurosurgery, Affiliated Hospital of Youjiang Medical University for Nationalities, Baise, China; ^5^ Department of Cardiothoracic Surgery, Affiliated Hospital of Nantong, University, Medical School of Nantong University, Nantong, China; ^6^ Department of Rehabilitation Medicine, Affiliated Hospital of Nantong University, Medical School of Nantong University, Nantong, China; ^7^ Department of Interventional Oncology, Renji Hospital, Shanghai Jiao Tong University School of Medicine, Shanghai, China

**Keywords:** cuproptosis, triple-negative breast cancer, tumor microenvironment, immunotherapy, tumor mutation burden

## Abstract

**Background:**

Cuproptosis is a copper-dependent cell death mechanism that is associated with tumor progression, prognosis, and immune response. However, the potential role of cuproptosis-related genes (CRGs) in the tumor microenvironment (TME) of triple-negative breast cancer (TNBC) remains unclear.

**Patients and methods:**

In total, 346 TNBC samples were collected from The Cancer Genome Atlas database and three Gene Expression Omnibus datasets, and were classified using R software packages. The relationships between the different subgroups and clinical pathological characteristics, immune infiltration characteristics, and mutation status of the TME were examined. Finally, a nomogram and calibration curve were constructed to predict patient survival probability to improve the clinical applicability of the CRG_score.

**Results:**

We identified two CRG clusters with immune cell infiltration characteristics highly consistent with those of the immune-inflamed and immune-desert clusters. Furthermore, we demonstrated that the gene signature can be used to evaluate tumor immune cell infiltration, clinical features, and prognostic status. Low CRG_scores were characterized by high tumor mutation burden and immune activation, good survival probability, and more immunoreactivity to CTLA4, while high CRG_scores were characterized by the activation of stromal pathways and immunosuppression.

**Conclusion:**

This study revealed the potential effects of CRGs on the TME, clinicopathological features, and prognosis of TNBC. The CRGs were closely associated with the tumor immunity of TNBC and are a potential tool for predicting patient prognosis. Our data provide new directions for the development of novel drugs in the future.

## Introduction

Breast cancer has surpassed lung cancer to become the most common cancer type worldwide. In 2020 alone, there were about over 2.3 million new breast cancer cases globally, accounting for approximately 11.7% of all tumors (1). Breast cancer is classified into four classical subtypes, Luminal A, Luminal B, HER2-enriched, and triple-negative breast cancer (TNBC), based on immunohistochemical (IHC) subtypes and comprehensive genetic analysis. TNBC accounts for about 15% to 20% of invasive breast cancers, and has high heterogeneity, invasiveness, and risk of recurrence (2, 3). In addition, it lacks specific targets and targeted therapeutic drugs, which are the key reasons for the observed failure of anticancer therapy and ultimate death of patients.

At present, systemic chemotherapy remains the standard treatment for TNBC (4). First-line chemotherapy consisting of anthracycline, taxane, and platinum drugs is used to treat high-risk, locally advanced TNBC. However, the rates of recurrence and distant metastasis remain high (5). The results of a Phase III clinical trial (KEYNOTE-355) comparing pembrolizumab combined with chemotherapy versus chemotherapy alone showed that for locally advanced/metastatic TNBC patients, pembrolizumab combined with chemotherapy only achieved significant progression-free survival (PFS) rates in the CPS 10 or higher subgroup, while the treatment in the remaining CPS subgroups had no significant effect (6). Another study in patients with locally advanced/metastatic TNBC (IMpassion131) showed that even with 45% of patients being PD-L1 positive, the combination of atezolizumab and paclitaxel did not improve the PFS of the patients (7). This suggests that determining a treatment method and estimating prognosis in patients based on PD-L1 expression alone are not accurate. Recently, increasing numbers of immune inhibitors have been approved for clinical use. However, patient survival and prognosis have not yet significantly improved, indicating that the optimal relationship between immune and chemotherapy drugs and TNBC patients has not been clarified. Therefore, a prediction model that can accurately characterize and classify the tumor microenvironment (TME) of patients is required for identifying patients who are sensitive to immune checkpoint inhibitors. Additionally, this would prevent patients who would not benefit from such drugs from undergoing unnecessary treatments.

Copper is an important metal element in organisms that participates in many biological processes, including mitochondrial respiration, iron absorption, oxidation resistance, and detoxification. The imbalance of copper homeostasis has been confirmed to be related to Menkes disease, Wilson disease, Alzheimer’s disease, blood diseases, metabolic syndrome, cardiovascular disease, and cancer (8). Copper is involved in the occurrence and development of malignant tumors by promoting cell proliferation, angiogenesis, and metastasis. Copper ions mediate the key steps of angiogenesis by binding to angiogenin and HIF-1. Furthermore, copper-induced oxidative stress can destroy DNA chains or modify molecular structures to activate oncogenes (9, 10). The latest research has revealed a previously unknown mechanism of cell death regulation, which has been named cuproptosis. Cuproptosis is a process mainly occurring in cells actively involved in respiration and the TCA cycle that promotes the combination of copper and fatty acylating component, resulting in the aggregation of fatty acylating protein, loss of iron-containing sulfur cluster protein, induction of HSP70, initiation of intracellular toxic oxidative stress, and eventually cell death (11). Studies have shown that copper is highly present in the serum or tumor tissues of patients with various cancers, including breast cancer, lung cancer, colorectal cancer, cervical cancer, ovarian cancer, and others. Additionally, copper is related to the occurrence, invasion, and metastasis of tumors (12–14). However, there are no reports describing any effects of the cuproptosis regulatory mechanism on TNBC. Therefore, to examine the role of cuproptosis in TNBC, we explored the possible prognostic value of cuproptosis-related genes (CRGs) in this study.

## Materials and methods

### Data sources

Data were obtained from the Gene Expression Omnibus (GEO; https://www.ncbi.nlm.nih.gov/geo/) and The Cancer Genome Atlas (TCGA) (https://portal.gdc.cancer.gov/) databases. RNA expression data were obtained for breast cancer from two GEO cohorts (GSE135565 and GSE65194) (15; 16) and from the TCGA cohort for subsequent analysis. We obtained the original “CEL” file and performed background adjustment and quantile normalization. In total, 253 cohort samples were included in the follow-up study. After excluding the samples with no prognosis and 0 and negative values for survival data, 239 samples were ultimately included in the follow-up study. Statistics were performed based on clinical variables such as tumor stage and grade, tumor location, patient age, gender, ethnicity, and prognosis. Additional samples (n=107) from GSE58812 (17) were included for further analysis in the construction of the CRG signature. The final included sample size was n=346. Batch effects from non-biological technical biases were corrected using the “ComBat” algorithm of the sva package (18; 19).

### Consistent clustering analysis and clinical feature comparison of CRGs

Sixteen genes related to cuproptosis were retrieved from the latest literature (11), of which 15 genes were expressed in the combined samples of TCGA and GEO. The “network” package was used to construct a gene network for these 15 CRGs. R “Cluster” (20) was used for consensus unsupervised clustering analysis, and the patients were classified into two subtypes according to CRG expression levels. After clustering, the correlation within the group was increased, while the correlation between groups was decreased. The prognoses of the two subgroups were compared using R “clusterSur”. We compared the relationships between subgroups and clinical pathological features, which included age, race, tumor location, T, N, M, and tumor staging. Subsequently, the associations of the subgroups with pathways and immunologic infiltration were compared.

### Genotyping of CRGs

To analyze the prognostic typing associated with CRGs in breast cancer, Cox regression analysis was used to evaluate the relationship between each gene in the combined TCGA and GEO datasets and survival state. In total, five CRGs with P<0.05 were screened out. The R “Cluster” package was used to divide the samples into groups A and B according to the expression of these five genes. To quantify the cuproptosis pattern of individual tumors using two prognosis-related genes based on CRGs, a gene cluster was established using the “NMF” package (21). This divided the patients into high and low groups C1 and C2, and analyzed the differences in survival and clinical characteristics between the two groups.

### Construction of a prognosis signature related to cuproptosis

Based on samples from two GEO and one TCGA datasets, we further included the LASSO Cox regression model and R “clusterSur” to narrow the range of candidate genes and establish a signature. The risk score was calculated as follows: Risk _ score =Σ (Expi * Coefi), where Coefi and Expi represent the risk factor and expression of each gene, respectively. The patients were divided into a training group (n =173), a test group (n=173), and a LOOCV verification group (n=3,462) with the propensity score matching model. According to the median risk scores, the three groups were divided into low-risk (CRG_score < median) and high-risk (CRG_score > median) groups. R “cluster SUR” was used for survival analysis and R “risk plot” and “ROC” were used for risk factor analysis and receiver operating characteristic curve analysis, respectively. Additionally, the survival states of the high- and low-risk groups were evaluated. Finally, time-dependent ROC curves for 1-, 3-, and 5-year survival were used to assess the ability of the signature to predict prognosis in the high- and low-risk groups.

### Clinical correlation and immune correlation of the prognostic CRG_score

The chi-square test was used to explore the relationships between CRG_score and clinical features (age, race, tumor location, T, N, M, and tumor stage). To assess whether the risk score was independent of other available clinical pathology features, univariate and multivariate analyses were performed on the training and testing sets. In addition, we performed a stratum analysis to determine whether the CRG_score retained its predictive power in the different subgroups. The immune status and stromal scores, microsatellite instability (MSI), dysfunction, exclusion, and tumor immune dysfunction and exclusion (TIDE) total scores between the high- and low-risk groups were displayed as a violin chart using the ESTIMATE algorithm (22). Furthermore, R “immunocor” was used to quantify the infiltration of immune cells in the high- and low-risk groups. To evaluate the proportion of tumor-infiltrating immune cells (TIICs) in the TME, CIBERSORT was used to quantify the abundance of multiple immune cell types in the two groups (23). We also explored the association of 22 infiltrating immune cell types with CRG_cluster and gene_Cluster in the high- and low-risk groups. We used Spearman’s analysis to examine differential expression levels at immune checkpoints between the subgroups.

### Mutation and drug sensitivity analysis

To determine the pattern of somatic mutations in TNBC patients in the high-risk and low-risk groups, a mutation annotation format (MAF) from the TCGA database was generated using the “MAF Tools” R software package. We also calculated the tumor mutation burden (TMB) score for each patient in both groups and performed a Kaplan-Meier survival analysis for H-TMB and L-TMB. To explore the drug sensitivity of the two groups of patients, the “pRRophetic” software package was used to calculate the semi-inhibitory concentration (IC50) values of TNBC after multi-drug treatment (24).

### Establishment and verification of nomogram scoring system

Using the results of the independent prognostic analysis, clinical features and risk scores were used to develop predictive nomograms using the “nomoR” package (25). In the nomogram scoring system, the total score was obtained by adding the scores of all the variables for each sample. The “timeroc” software package was used for ROC curve analysis of the 1-, 3-, and 5-year survival rates (26; 27). Calibration plots of nomograms are used to describe the predicted values between the predicted 1-, 3-, and 5-year survival events and the actual observed results.

### Cell culture and transfection

The two human breast cancer cell lines (MDA-MB-231 and BT-549) were representative in cancers studies, and were obtained from the Cell Bank of Shanghai Institutes of Biological Sciences, Chinese Academy of Sciences (Shanghai, China). The MDA-MB-231 and BT-549 cells were cultured in RPMI 1640 medium (Gibco, CA, USA) with 10% fetal bovine serum (Gibco, USA) and 1% penicillin-streptomycin solution (Gibco, CA, USA) at 37°C in a humidified incubator containing 5% CO2. The medium was refreshed every 2 days.

Small interfering RNA (siRNA) knockdown of ATP7A Transient silencing of the ATP7A gene was achieved using a pool of siRNA duplexes (ONTARGETplus SMARTpool, Dharmacon). The siRNA sequences were as follows:

si-Ctrl: 5’ - CAA GAG UUA CAA UAG UUG C - 3’,
siRNA-1: 5’ - UAU CCU AUG GUU AAA CCU CUG - 3’,
siRNA-2: 5’- GCA ACU AUU GUA ACU CUU G -3’.

The siRNA was transfected into indicated cells using using Lipofectamine 3000 (Invitrogen; Thermo Fisher Scientific, Inc., USA) according to the manufacturer’s instructions, followed by Western bloting assay to verify the efficacy of interference.

### Western blotting

The total proteins from breast cancer cells were extracted using a Total Protein Extraction kit (#C006225; Sangon Biotech) according to the manufacturer’s instructions. Briefly, the protein samples were then isolated using 12% SDS-PAGE (sodium dodecyl sulphate-polyacrylamide gel electrophoresis; Sangon Biotech), and the protein bands were transferred onto PVDF (polyvinylidene fluoride) membranes, which were subsequently blocked with 5% lipid-free milk solution. The membranes were then incubated overnight with diluted primary antibodies at 4 °C, and incubated for 2 h with diluted secondary antibodies at room temperature, and finally developed with ECL Western Blotting Substrates (#32109; Thermo Fisher Scientific). The primary antibodies used for Western blotting were anti-ATP7A (#ab13995; Abcam) and anti-GAPDH (#5174; CST).

### Cell proliferation and migration assays

The stably transfected MDA-MB-231 and BT-549 were divided into different groups and seeded onto a 96-well plate at a density of 2×10^4^ cells/ml. Next, the Cell Counting Kit-8 (CCK-8 Kit; Dojindo, Japan), based on the manufacturer’s instructions, was added to determine the proliferative capacity of cells. Optical density (OD) values were obtained at 450 nm and was measured at 1, 2, 3, 4 and 5 days after seeding using an automatic microplate reader (TEAN, Swiss). Three replicate analyses were performed for each sample. A transwell cell migration assay was used to test the ability of cells to metastasize. The cell density of different groups was adjusted to 2×10^5^ cells/ml, and 100 μl cell suspension of different groups were added to the upper chamber with or without Matrigel (Corning, USA). The medium containing 20% fetal bovine serum was added in the lower 24-well plate chamber. After 24 h, the bottom MDA-MB-231 and BT-549 cells were treated with 4% polyoxymethylene for 15 min, deionized water, and 0.1% crystal violet for 30 min. Finally, the MDA-MB-231 and BT-549 cells migrating to the lower surface of transwell chamber were counted using a microscope in six random fields utilizing a 200x microscope.

### Statistical analysis

R survival package was used for survival analysis, and the survival rate of each group was tested by Log-Rank test hypothesis. Kruskal-Wallis test is used to compare two or more groups of data, and Wilcoxon test was used to compare two groups of data. Kaplan-Meier method was used to generate the survival curve of each subgroup in the data set. Chi-square test was used to analyze the mutation frequency between ICI score subgroup and somatic cells, and Spearman analysis was used to calculate the correlation coefficient. All statistical analyses were performed using R version 4.1.0. The statistical significance was set to P<0.05.

## Results

### Landscape of genetic variation of CRGs in breast cancer

**Figure 1:**
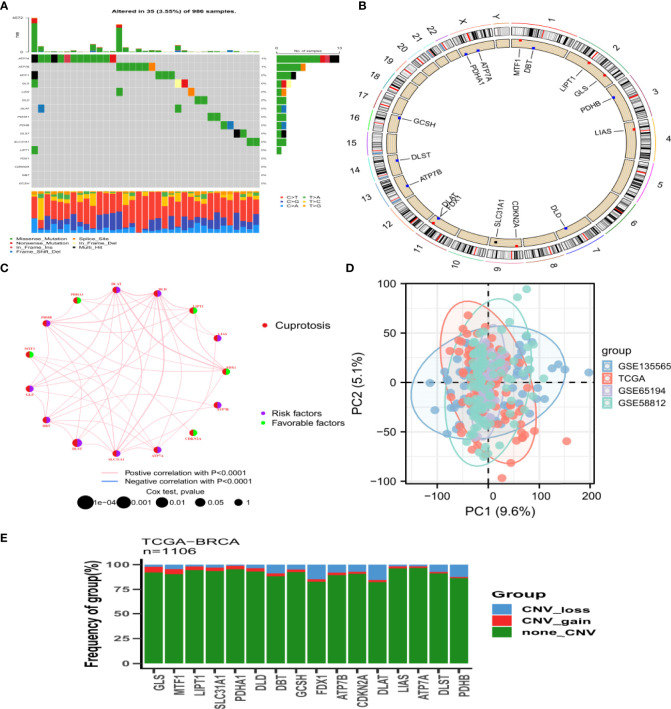
Landscape of genetic variation of Cuprotosis related genes in BRCA **(A)** Mutation frequency of 16 CRGs in 986 BRCA from TCGA and GEO combined samples. The number on the right indicated the mutation frequency of each gene. The bar chart on the right showed the proportion of mutations. The stacked bar chart below shows the fraction of conversions. **(B)** The position of CRGs CNV changes on 23 chromosomes. **(C)** Interaction between CRGs in BRCA. The line connecting CRGs indicated their interaction, and the thickness of the line indicated the correlation strength between CRGs. Purple and green represent negative and positive correlation respectively. **(D)** The figure of the PCA of the TCGA and GEO (GSE13356, GSE65194, GSE58812) datasets. **(E)** CNV mutation frequency of CRGs. The deletion frequency, blue dot; The amplification frequency, red dot. BRCA, breast cancer; CRGs, cuprotosis related genes; CNV, copy number variant.

In this study, 16 genes related to cuproptosis were identified in the literature. We first summarized the incidence of copy number variation (CNV) and somatic mutations of these CRGs in breast cancer. Only 35 of 986 samples (3.55%) showed genetic variation, and in the mainly mutated genes ATP7A and ATP7B, the frequency was only 1% (**Figure 1A**). **Figure 1B** shows the location of the CNV changes on the chromosomes for the CRGs, with the CNV changes occurring universally for 16 genes. Further investigation of the frequency of CNV mutations revealed that CNV amplification was more frequent in GLS and MTF1, whereas the FDX1, DLAT, and PDHB genes were more frequently expressed as CNV deletions (**Figure 1E**). For these 16 genes, we constructed an interaction network diagram of CRGs to demonstrate their interactions (**Figure 1C**). Furthermore, principal component analysis (PCA) showed that the data in the four cohorts had reduced dispersion after sample normalization (**Figure 1D**). The above analysis indicates that the landscape of genetic and expression changes of CRGs in breast cancer was highly heterogeneous, revealing that the imbalance of CRG expression plays a key role in the occurrence and development of breast cancer.

### Enrichment of five prognosis-related CRGs

**Figure 2:**
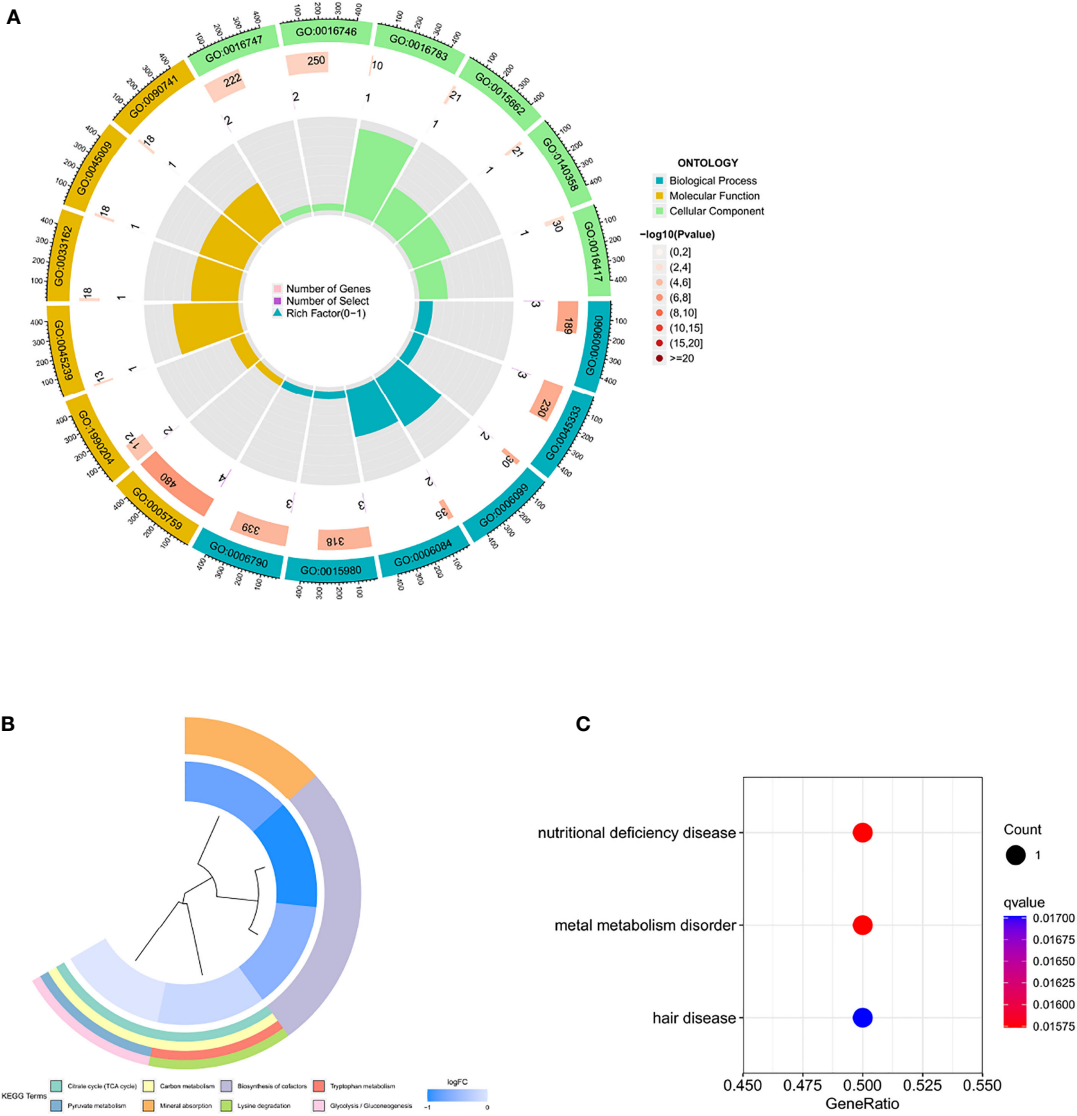
Enrichment of five prognosis-related CRGs **(A)** Gene ontology enrichment **(B)** KEGG metabolic pathway Enrichment **(C)** Disease enrichment.

We analyzed the association between prognosis and CRG expression levels in patients, finding that the differential expression of five genes was significantly correlated with prognosis. Among them, the high expression levels of ATP7A, DLST, and LIAS were associated with poor overall survival (OS), while the high expression levels of LIPT1 and PDHA1 suggested a good prognosis (**Figure S4**). Gene ontology enrichment analysis of the five differentially expressed genes (DEGs) revealed that these genes were mainly involved in biological processes and molecular functions, and less in cellular components (**Figure 2A**). Subsequently, KEGG metabolic pathway analysis showed that the five DEGs were significantly enriched in the tricarboxylic acid cycle, which possibly affects the occurrence of the disease by negatively regulating the metabolic function of mitochondria (**Figure 2B**). Finally, disease enrichment analysis showed that diseases such as nutritional deficiency, hair disease, and metal metabolism disorders were associated with CRGs (**Figure 2C**).

### Tumor classification and related immune infiltration based on CRGs

**Figure 3:**
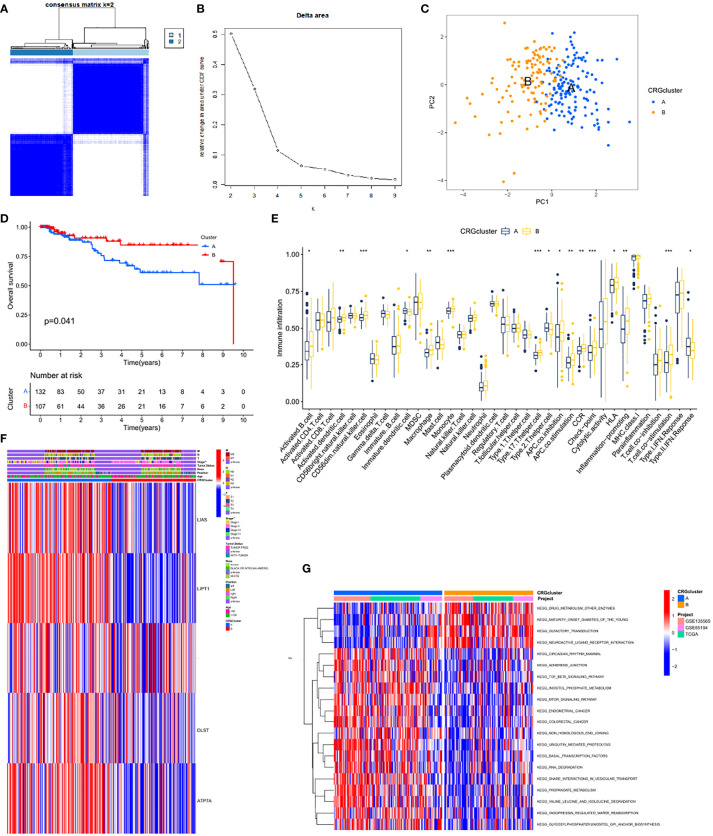
CRG subtypes and clinicopathological and biological characteristics of two distinct subtypes of samples divided by consistent clustering. **(A)** Consensus matrix heatmap defining two clusters (k = 2) and their correlation area. **(B)** relative change area under cumulative distribution function curve. **(C)** PCA analysis revealed significant differences in transcriptome between the two subtypes. **(D)** Kaplan-Meier curve showed that there were significant survival differences between cluster A and B (P=0.041). **(E)** The abundance of each TME infiltrating cell in two clusters. The upper and lower ends of the boxes represented interquartile range of values. The lines in the boxes represented median value, and black dots showed outliers. The asterisks represented the statistical p value (*P < 0.05; **P < 0.01; ***P < 0.001). **(F)** Differences in clinicopathologic features and expression levels of CRGs between the two distinct subtypes. **(G)** GSVA of biological pathways between two distinct subtypes, in which red and blue represent activated and inhibited pathways, respectively.

To further elucidate the clinical value or functional biological pattern of these CRGs, we performed consistent clustering and grouped the TCGA TNBC cohort samples into subgroups based on the expression levels of 16 CRGs. We found that when K=2, we can provide the best clustering stability from k=2 to k=9. The TCGA TNBC cohort was categorized according to two different CRGs (**Figures 3A, B**) into CRG cluster A (n=132) and CRG cluster B (n=107). Additionally, based on the expression levels of CRGs, PCA revealed significant differences in the transcriptional profiles of CRGs between the two groups (**Figure 3C**). Analysis of TME immune infiltration for TNBC showed that the CRG cluster B group was rich in innate immune cell infiltration, including activated B cells, activated dendritic cells, CD56dim natural killer cells, macrophages, and others (**Figure 3E**). According to the heat map, CRG cluster A group showed increased expression levels of LIAS, LIPT1, DLST, and ATP7A. Notably, there was a significant difference in tumor stage between the two groups (P<0.01, **Figure 3F**). To explore the biological behaviors of these CRGs, we performed a gene set variation analysis. As shown in **Figure 3G**, CRG cluster A is significantly enriched in stroma and oncogenic activation pathways such as the TGF- signaling pathway, adhesion, phosphoinositide metabolic pathway, and mTOR signaling pathway. Further survival analysis showed that CRG cluster B had better OS for patients than cluster A (**Figure 3D**). Cluster A showed a matching survival disadvantage. In summary, CRG cluster A can be classified as an immune-desert phenotype with abundant stroma and oncogenic signaling pathways, while CRG cluster B was classified as an immune-inflamed phenotype characterized by innate immune cell infiltration and immune activation.

### Generation and functional annotation of CRGs

**Figure 4:**
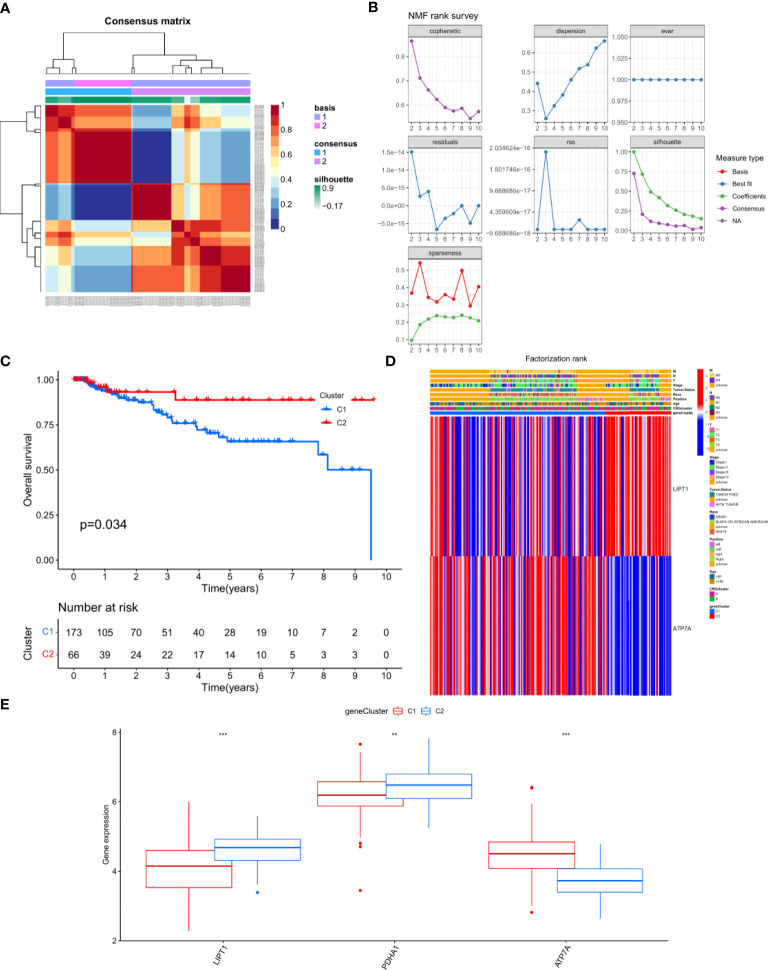
Generation and functional annotation of CRGs **(A, B)** Two subgroups was identified the optimal value for consensus clustering,and was designated genecluster 1 (C1) and genecluster 2 (C2). **(C)** Survival curve of the patients between C1 and C2(P = 0.034). **(D)** Differences in clinicopathologic features and expression levels of CRGs between the C1 and C2. **(E)** The expression of 3 CRGs between C1 and C2. The asterisks represented the statistical p value (**P < 0.01; ***P < 0.001).

To further analyze the genetic characteristics of CRGs, we used NMF clustering analysis to separate the patients into different gene subtypes. Consistent with CRG classification, TNBC patients were divided into two gene subgroups, designated C1 (n=173) and C2 (n=66, **Figures 4A, B**). We found that T4, M1, N2-3, and Stage VI in tumor staging were mainly concentrated in the C1 group and were confirmed to be associated with worse OS (**Figures 4C, D**). The analysis also revealed that the two groups had different genetic characteristics. ATP7A was mainly expressed in the C1 group, while LIPT1 and PDHA1 were significantly enriched in the C2 group (**Figure 4E**).

### Construction of the prediction model

**Figure 5:**
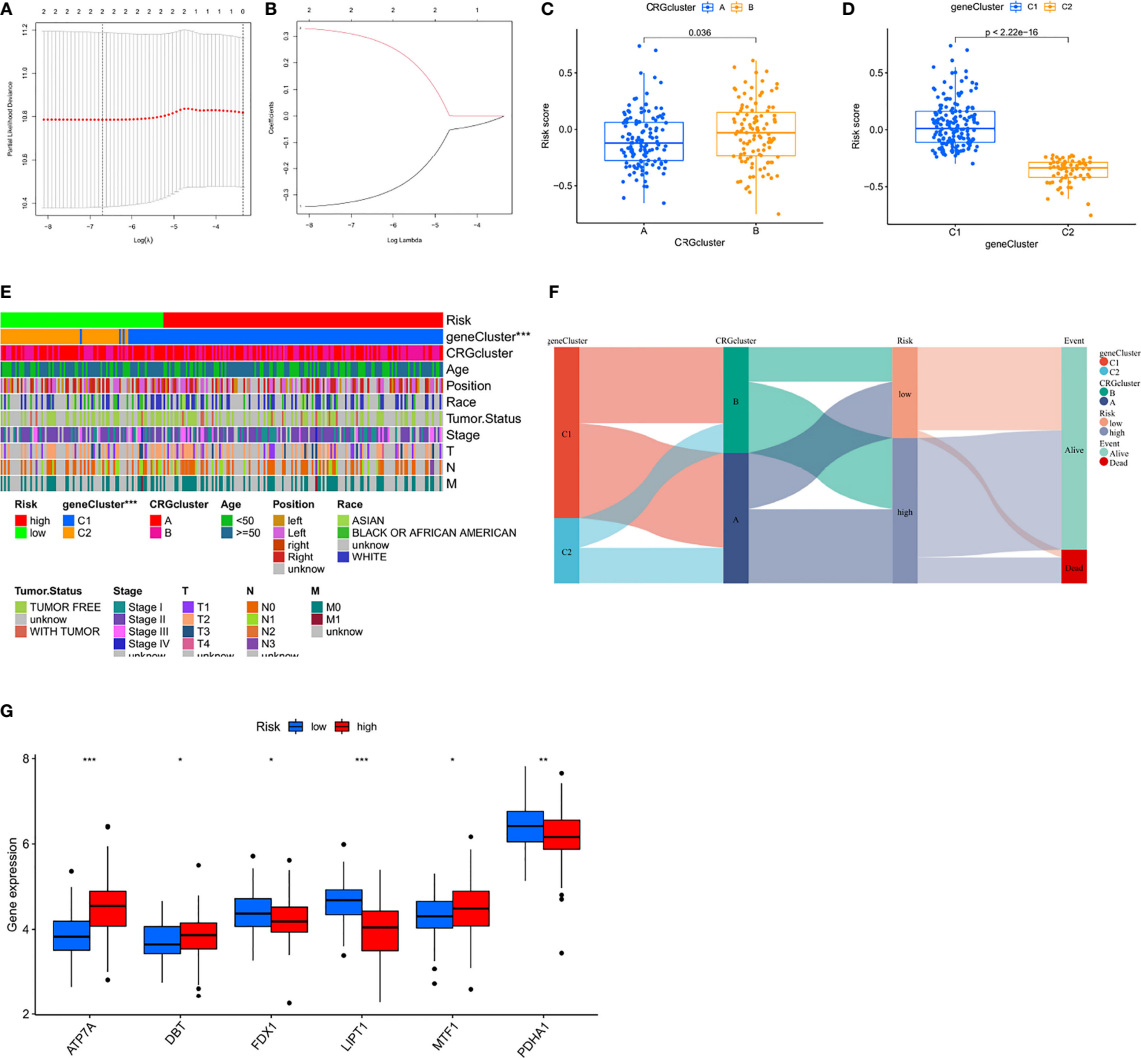
Construction of the prognosis risk prediction model **(A, B)** The least absolute shrinkage and selection operator (LASSO) regression was performed with the minimum criteria. **(C, D)** Differences in CRG_score between gene subtypes and cuprotosis subtypes **(E)** Differences in clinicopathologic features and expression levels of CRGs between the high-and low-risk group. **(F)** Alluvial diagram of subtype distributions in groups with different CRG_scores and survival outcomes. **(G)** Six differential expressions of CRGs in high and low risk groups. The asterisks represented the statistical p value (*P < 0.05; **P < 0.01; ***P < 0.001).

The above study used a population analysis approach, making it difficult to accurately predict the CRG pattern in patients on an individual basis. Given the individual heterogeneity and complexity of TNBC patients, we attempted to determine the prognostic gene set for TNBC using the LASSO method. A prognostic model was established using the abovementioned genes, and a gene model capable of quantification in each patient was created using a regression model with a minimum lambda (**Figures 5A, B**). With these, we constructed a scoring system to quantify the CRG pattern in a single patient, which we called the CRG_score. Subsequently, we used the Kruskal-Wallis test to verify the relationship between the CRG_score and CRG cluster. Surprisingly, the results showed that the high score group matched with CRG cluster B and the low score group matched with CRG cluster A (**Figure 5C**). In the subgroup comparison of CRG_score and gene cluster, we found that the CRG_score of C1 was higher than that of C2, which was consistent with our expectation (**Figure 5D**). Next, we performed subgroup clustering using unsupervised clustering and found a significant statistical difference between CRG_score and gene cluster (P<0.001, **Figure 5E**). To better visualize the attributes of individual patients and find any correlations with survival, we used the alluvial diagram and found that patients with low scores were all surviving, while some patients with high scores had died (**Figure 5F**). Significant expression of genes related to the regulation of cuproptosis was observed in both CRG gene clusters and was consistent with the expected results for a subset of CRGs (**Figure 5G**).

### Prognosis value of the risk model in the training, test, and entire sets

**Figure 6:**
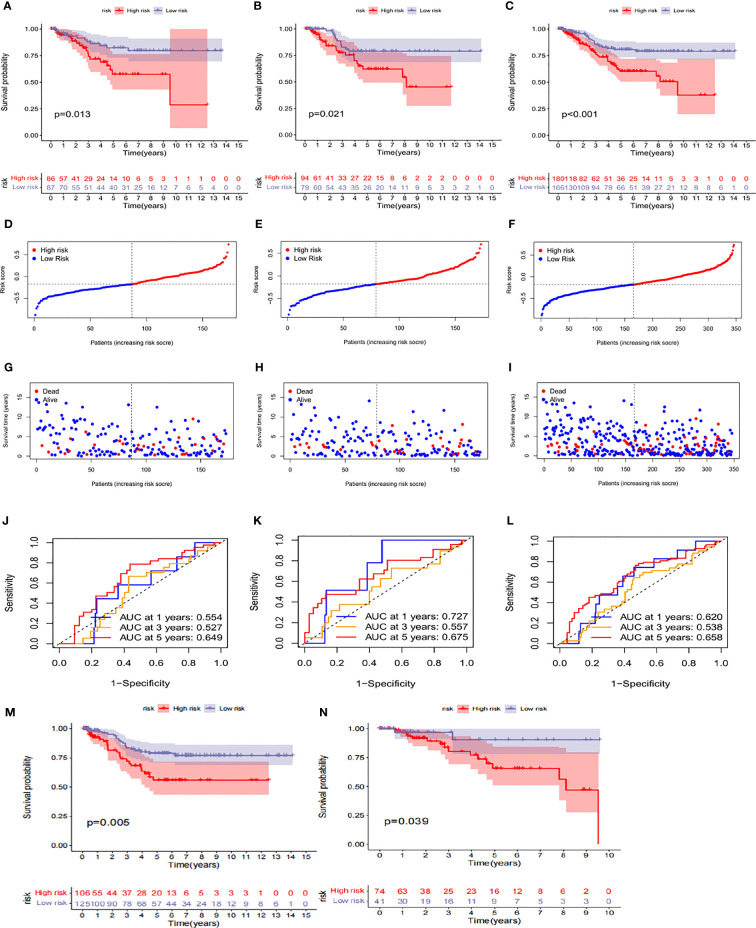
Prognosis value of the risk model in the train, test, and entire sets **(A–C)** Kaplan–Meier survival curves of survival probability of patients between low-and high-risk groups in the train,test, and entire sets, respectively. **(D–F)** Exhibition of CRGs model based on risk score ofthe train, test, and entire sets, respectively. **(G–I)** Survival time and survival status between low-and high-risk groups in the train, test, and entire sets, respectively. **(J–L)** ROC curves to predict the sensitivity and specificity of 1-, 3-, and 5-year survival according to the CRG_score in the train,test, and entire sets, respectively. **(M, N)** Survival probability in the high-risk and low-risk subgroups of the GEO cohort and TCGA cohort, respectively.

The risk score was calculated according to the following formula: Risk score = (-0.31657 * LIPT1) + (0.29823 * ATP7A). The survival probability, risk score distribution, survival status, and related gene expression of patients in the low and high subgroups were compared in the three cohorts of Training Set A (n=173), Verification Set B (n=173), and LOOCV Verification Set C (n=3462). Overall, these analyses indicated that the high subgroup has a poorer prognosis (**Figures 6A–I**). The time-dependent ROC curve was plotted and the AUC was calculated at different time points to estimate the performance of the predictive model. As shown in **Figures 6J–L**, AUC values for sets A, B, and C were 0.554, 0.727, and 0.620, respectively, at 1 year, 0.527, 0.557, and 0.538, respectively, at 3 years, and 0.649, 0.675, and 0.658, respectively, at 5 years. These data indicate that the predictive model had good predictive value for both short-term and long-term follow-up. After comprehensively considering the sample size and source platform of the cohorts, we analyzed the survival probability of GEO cohort (n=231) and TCGA cohort (n=115) (**Figures 6M, N**), and the results were also very exciting. At the same time, we subclassified all the TCGA and GEO databases involved in the analysis, namely TCGA, TCGA-non-tnbc, GSE58812 and Metabric, respectively. By analyzing the different effects of CRG on the survival of each subgroup in detail, it was indeed found that CRG had a unique effect on TNBC (p<0.05, **Figure S1**).

### Independence detection of the constructed risk prediction model

**Figure 7:**
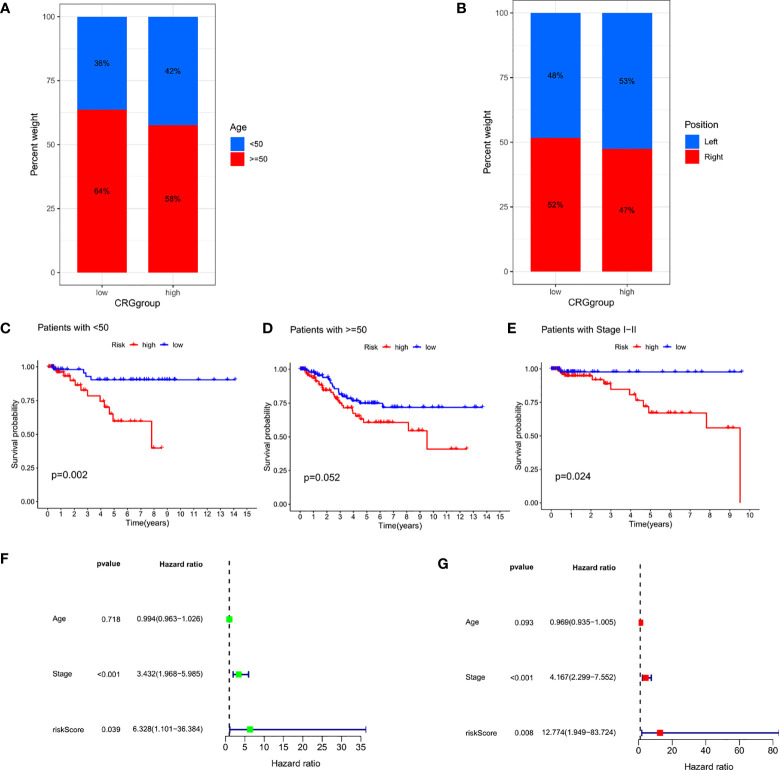
Independence detection of the constructed risk prediction model **(A)** Relationships between CRG_score and Age. **(B)** Relationships between CRG_score and Position. **(C–E)** Kaplan–Meier survival curves of survival probability prognostic value stratified by age and stage. **(F, G)** Uni- and multi-Cox analyses of clinical factors and risk score with OS.

In clinical correlation analysis, we found that high CRG_scores were observed more in young women, and there was no significant difference in tumor location between the two groups (**Figures 7A, B**). Univariate and multivariate Cox regression analyses including the patient’s age and tumor stage confirmed that tumor stage and CRG_score were independent prognostic factors for OS in TNBC patients (**Figures 7F, G**). In addition, our stratification analysis confirmed that CRG_score also had age-independent prognostic value (**Figures 7C–E**). Therefore, we believe that CRG_score is a reliable and independent prognostic biomarker for evaluating the prognosis of TNBC.

### Comparison of immune activity between subgroups

**Figure 8:**
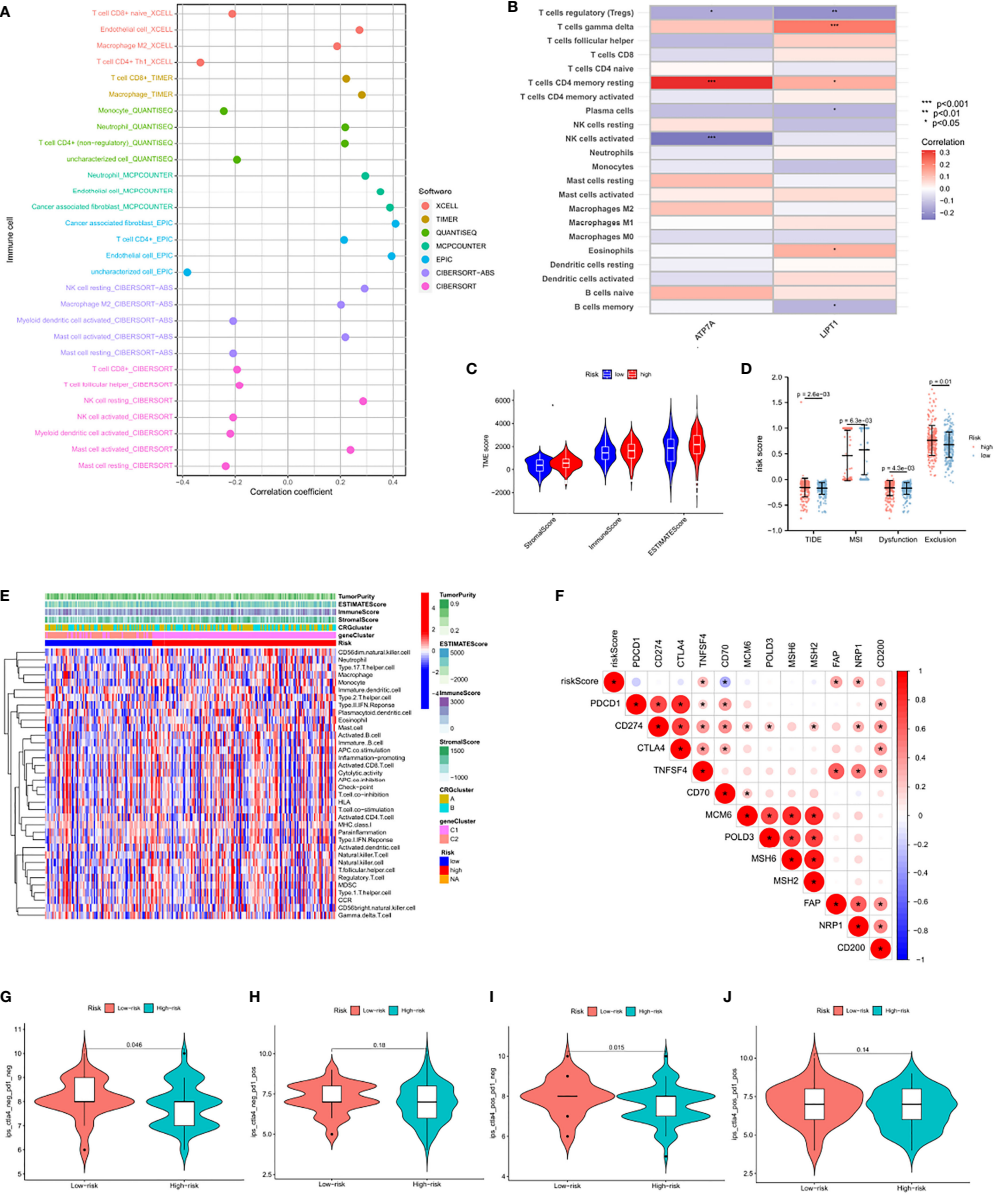
Comparison of immune activity between subgroups **(A)** The immune cell bubble of risk groups **(B)** Correlations between the abundance of immune cells and 2 genes in the proposed model. **(C)** Correlations between CRG_score and both immune and stromal scores. **(D)** Correlations between CRG_score and TIDE, MSI, Dysfunction and Exclusion. **(E)** Differences in immune scores, stromal scores and expression levels of immune cells between the high-and low-risk group. **(F)** Correlation of 12 immune checkpoint gene risk scores. **(G–I)** The association between IPS and the CRGs based on TCIA database, **(G)** CTLA4– PD1–**(H)** CTLA4– PD1+ **(I)** CTLA4+ PD1– **(J)** CTLA4+ PD1+. The asterisks represented the statistical p value (*P < 0.05; **P < 0.01; ***P < 0.001).

On different platforms shown in the immune cell bubble diagram (**Figure 8A**), we found that more endothelial cells and cancer-associated fibroblasts (CAFs) were associated with the high-risk group. CAF activation has been confirmed to be strongly involved in cancer progression through their complex interactions with other cell types in the TME (28). **Figure 8B** shows the correlation between immune cells and genes: gamma delta T cells, CD4 memory resting T cells, and eosinophils have significant positive correlations with LIPT1, while plasma cells and memory B cells have significant negative correlations with LIPT1. CD4 memory resting T cells showed a significant positive correlation with ATP7A, while regulatory T cells and activated natural killer (NK) cells showed significant negative correlations with ATP7A. The association of these genes with immune cells was as expected. In addition, stromal activation in the TME was generally considered to be T-cell inhibitory. TME scores performed on different subgroups indicated that the high score group had higher tumor stromal scores (**Figure 8C**). Stromal activation would likely result in immunosuppression and worse prognosis in the high score group. Furthermore, according to the comprehensive map of TNBC, four immune scores were given, including TIDE, Dysfunction, Exclusion, and MSI. We assessed the differences in CRG_score between these molecular subtypes. High CRG_scores were found in TIDE, Dysfunction, and Exclusion, and patient prognosis was poor. However, the low score group had a higher MSI, representing a better prognosis (**Figures 6** and **8D**).

Previous studies have shown that Dysfunction and Exclusion, as the two major mechanisms of tumor immune escape, can accurately predict the effect of immunotherapy. However, the TIDE score, a more optimized immune simulation calculation method, can stably predict the effect of immunotherapy (29). Detection of tumor MSI has been widely recognized as a means of predicting tumor immune response in pan-cancerous species. Given that the CRG_score was highly consistent with these four tumor immunopredictors, the CRG_score may be a more effective biomarker for predicting the efficacy of immunotherapy than the above four scores. **Figure 8E** showed the differences in immune scores, stromal scores and expression levels of immune cells between the high-and low-risk group. Interestingly, the threshold for Fisher’s exact test was set to P<0.05, and the three common immune checkpoint genes PDCD1, CD274, and CTLA4 failed to show differences between the two subgroups (**Figure 8F**). We further explored the ICI treatment response represented by CTLA-4/PD-1 inhibitors. The patients in the CRG_score group showed significant therapeutic effects only with anti-CTLA-4 therapy (**Figure 8G–J**), which may partly explain the mismatch between the current TNBC immune targets and the immunotherapeutic effects.

Next, we performed a single-sample gene set enrichment analysis for all CRGs, and found that most of the genes were related to immunity, and then analyzed the role of CRG in BRCA. It can also be clearly found that CRG has played an important role in the immune microenvironment of BRCA. (**Figure S2**).

### Genetic characteristics of CRG_score and tumor somatic mutation of TNBC

**Figure 9:**
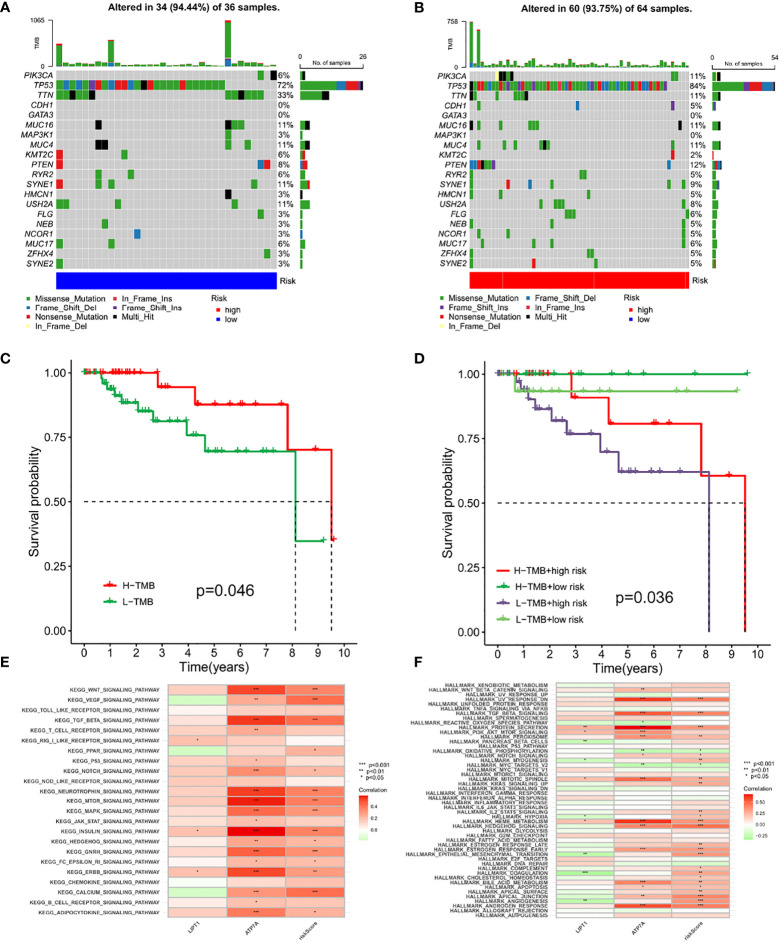
Genetic characteristics of CRG_score and tumor somatic mutation of TNBC **(A, B)** The waterfall plot of tumor somatic mutation established by those with high-and low-risk group. **(C)** The overall survival of H-TMB and L-TMB using Kaplan–Meier in Log-rank test. **(D)** The overall survival of the patients stratified by both the CRG-score signature and TMB using Kaplan–Meier curves. **(E, F)** Correlations between CRGs and Pathway Through KEGG and HALLMARK Enrichment Analysis. The asterisks represented the statistical p value (*P < 0.05; **P < 0.01; ***P < 0.001).

We then used the maftools package to analyze any difference in the distribution of somatic mutations for low and high CRG_scores in the TCGA-TNBC cohort. As shown in **Figures 9A, B**, the TMB was more extensive in the low score group. The most obvious somatic mutations in the low score group were TP53 (72%) and TTN (33%), while the most obvious somatic mutations in the high score group were TP53 (84%) and PTEN (12%). Evidence suggests that patients with H-TMB may benefit from immunotherapy. Further survival analysis demonstrated that the H-TMB subgroup showed significant survival benefits. The subgroup with both low CRG_score and H-TMB showed better survival benefits, while the subgroup with both high CRG_score and L-TMB had lower survival probability (**Figures 9C, D**). Next, we analyzed the correlation between CRG and CRG_score subgroups through the enrichment of the HALLMARK pathway and KEGG pathway (**Figures 9E, F**). The results showed that ATP7A and CRG_score, which were high in the C1 group, were consistent with the enrichment of signaling pathways in most cancers.

We performed KEGG analysis on all genes to analyze the association of CRGs with different pathways and mechanisms. We found that in Biological Process, it is mainly related to tricarboxylic acid cycle, citrate metabolic process, and acetyl-CoA metabolic process; In Cellular Component, it is mainly related to mitochondrial matrix, oxidoreductase complex, dihydrolipoyl dehydrogenase complex; In Molecular Function, it is mainly related to oxidoreductase activity, acting on the aldehyde or oxo group of donors, NAD or NADP as acceptor, oxidoreductase activity, acting on the aldehyde or oxo group of donors, transition metal ion transmembrane transporter activity. We also analyzed the KEGG pathway and found that CRG is mainly involved in the Citrate cycle (TCA cycle), Carbon metabolism, Pyruvate metabolism, Glycolysis/Gluconeogenesis, and Platinum drug resistance(**Figure S3**).

### Construction and evaluation of a nomogram based on CRG_score

**Figure 10:**
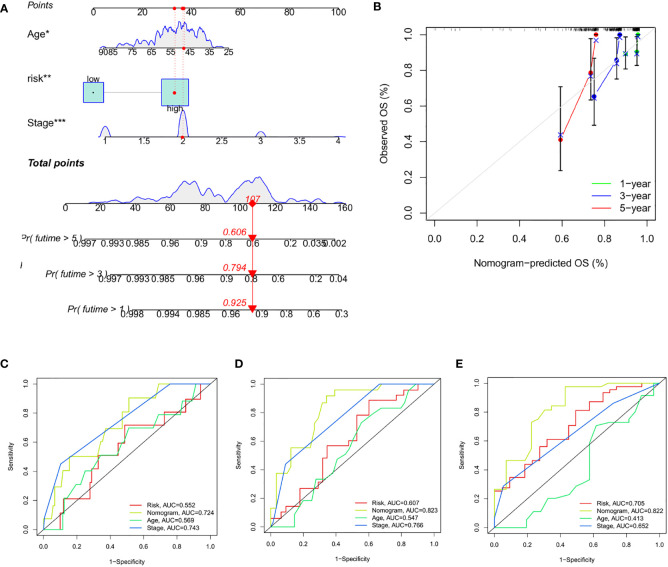
Construction and Evaluation of Nomogram Based on CRG_score **(A)** Nomogram for predicting the 1-, 3-, and 5-year OS of TNBC patients. **(B)** Calibration curves of the nomogram. **(C–E)** ROC curves for predicting the 1-, 3-, and 5-year ROC curves in the train, test, and entire sets. The asterisks represented the statistical p value (*P < 0.05; **P < 0.01; ***P < 0.001).

To quantify the individual risk assessment in TNBC patients, we developed a personalized score nomogram using three parameters, age, tumor stage, and CRG_score, to predict OS. Each arrow shows an example (**Figure 10A**). According to the scores of each prognostic index, the higher the total score was, the worse the clinical prognosis would be. Furthermore, the calibration plot shows that the nomogram operated in accordance with the ideal model (**Figure 10B**). The ROC curve was used to calculate the AUC value at different time points to evaluate the prediction performance of the nomogram model. As shown in **Figures 10C–E**, the AUC values for 1-year, 3-year, and 5-year were 0.724, 0.823, and 0.822, respectively. This indicates that the new nomogram prediction model has high accuracy.

### Comparison of anticancer drug sensitivity between patients with different CRG_scores

**Figure 11:**
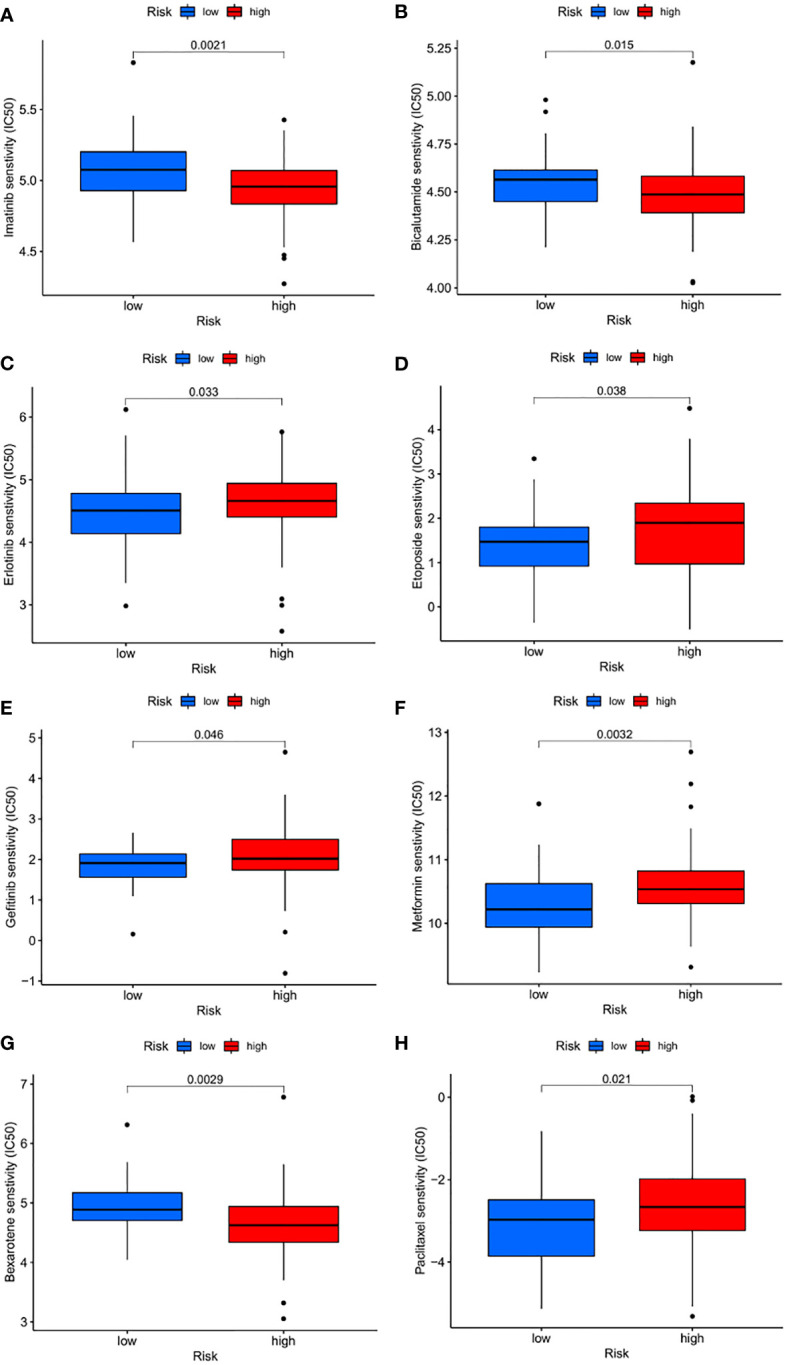
Drug sensitivity **(A–H)** Estimated drug sensitivity in patients with high and low FRLM risk.

We next selected anticancer drugs to assess the susceptibility of the low-and high-risk populations to these drugs. Interestingly, we found that the IC50 values of erlotinib, etoposide, gefitinib, and metformin were lower in patients with high CRG_scores. However, in patients with low CRG_scores, the IC50 values of chemotherapeutic agents such as imatinib, bicalutamide, and belotinib were significantly reduced. Taken together, these results suggest that the CRGs are associated with drug susceptibility (**Figures 11A–H**).

### Inhibition of ATP7A restrains proliferation and migration capacities of breast cancer cells

**Figure 12:**
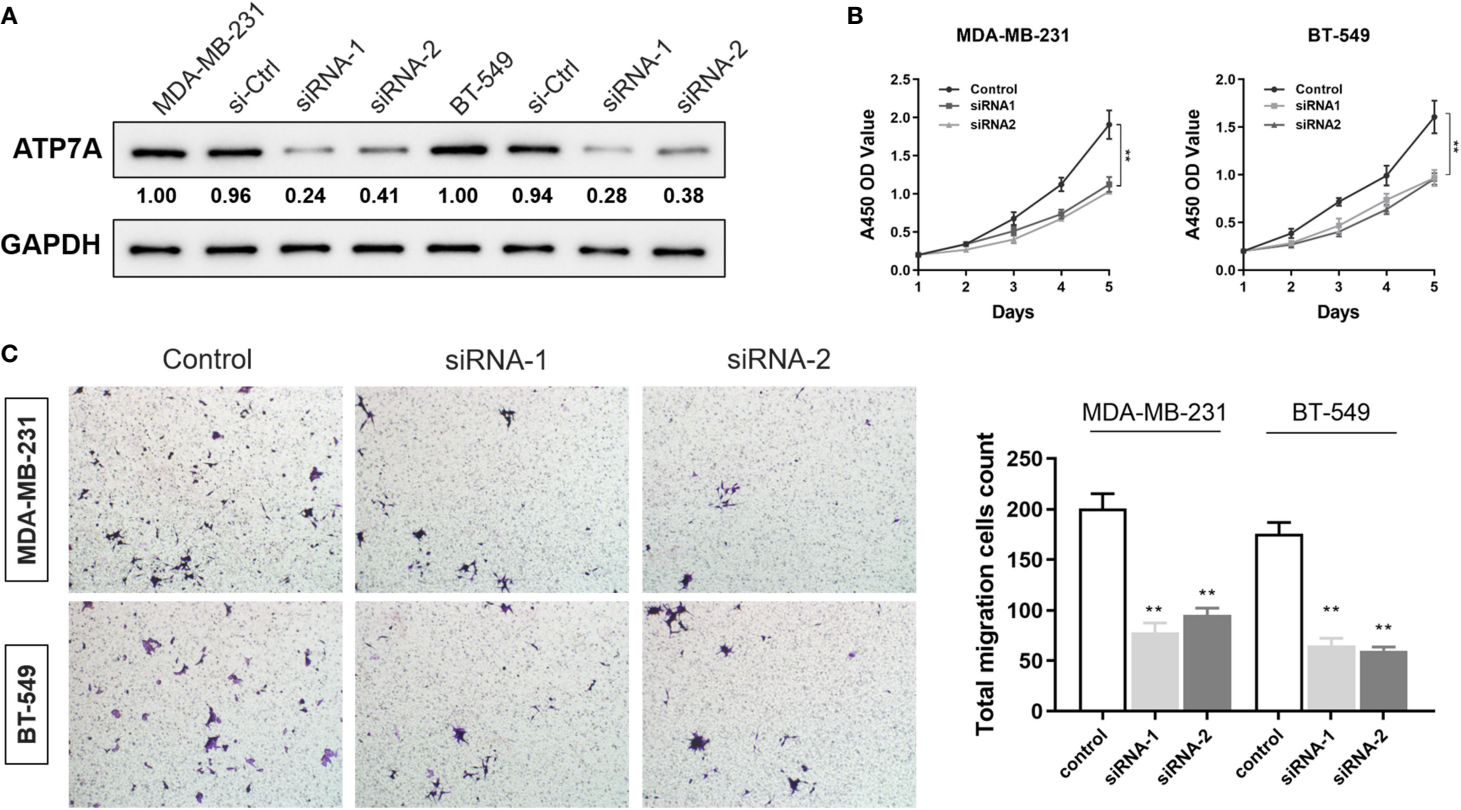
Inhibition of ATP7A restrained proliferation and migration capacities of breast cancer cells **(A)** Western blot analysis revealed the efficiency of ATP7A knocking-down in BT549 and MDA-MB-231 cell lines. **(B)** CCK-8 cell proliferation assay after ATP7A knockdown in BT549 and MDA-MB-231 cell lines. **(C)** The Transwell invasion assay showed that knocking-down of ATP7A inhibited the cellular invasion of the BT549 and the MDA-MB-231.Graphical representation of the number of invasive BT549 and MDA-MB-231 cells per microscopic field. Data were shown as the mean ± SD from three independent experiments. **P < 0.01, *vs*. control group.

To investigate malignant biological behaviors of ATP7A *in vitro*, we performed cellular experimental analysis, and validation down-regulated of ATP7A in BT-549 and MDA-MB-231 cells (**Figure 12A**). Using the CCK-8 assay, we found down-regulated expression level of ATP7A significantly suppressed the proliferative ability of breast cancer cells (**Figure 12B**). Transwell cell migration assay revealed significantly decreased migrated cell numbers in ATP7A-knockdown breast cancer cells compared with control group (**Figure 12C**). Taken together, the down-regulation of ATP7A expression markedly restrained proliferation and migration capacities of breast cancer cells, which is in consistent with its pro-tumorigenic role for patients with breast cancer.

## Discussion

Because of the dual role of metallic copper in tumor progression, this area is becoming a hot research topic in cancer biology. Copper is a valuable coenzyme in the metabolism of organisms (30), and the imbalance of copper ion homeostasis in the human body is also closely related to various diseases (31). Increasing evidence has shown that copper plays vital roles in tumor cell proliferation, angiogenesis, and cancer metastasis (32, 33). In addition, copper content levels in serum and tumor tissues of patients with various tumors, including TNBC, are often significantly increased. Serum copper levels can help predict the survival probability of patients with early TNBC (34, 35). Copper can induce cell death through apoptosis and the autophagy pathway. The application of copper-chelating agents, such as tetrathiomolybdate, has also achieved great efficacy in early clinical experiments in a variety of tumors. In addition, copper is widely used in many fields such as chemodynamic therapy, chemotherapy, phototherapy, and biological imaging (33). Recently, Peter Tsvetkov found a mitochondrial protein aggregation caused by excessive copper, a cell death mechanism called cuproptosis. Accumulating evidence suggests that cuproptosis may play indispensable roles in the TME, inflammation, and tumor immunity. Therefore, characterizing the global response mediated by multiple CRGs and TME cell infiltration may help identify potential prognostic features and determine immunotherapy strategies for TNBC.

The results of this study revealed overall changes in CRGs at both the genetic and transcriptional regulatory levels in TNBC patients. First, we identified two subtypes based on 16 CRGs, designated CRG cluster A and CRG cluster B. Surprisingly, the two subtypes had markedly different immune infiltration features and tumor signaling pathways. CRG cluster A, from a lack of an immune cell presence, had more of an immune-desert phenotype and was enriched in a variety of tumor signaling pathways, including the TGF- signaling pathway, adhesion, phosphoinositide metabolic pathway, and mTOR signaling pathway. CRG cluster B, however, was more in line with an immunoinflammatory phenotype because of the large degree of immune cell infiltration in the TME (36). This was consistent with our finding that the cluster A group patients had better prognoses compared with cluster B. Next, we identified two gene subtypes, C1 and C2, based on CRG expression between tumors. Late tumor stages and poor OS were also observed in C1. Therefore, a comprehensive evaluation of CRGs and clinical features not only helped to establish an accurate prognosis model, but also helped clinicians to effectively evaluate the rationality of immunotherapy as a treatment plan by depicting the TME through CRGs. Considering the individual heterogeneity and complexity of TNBC (37), a scoring mechanism that can quantify the characteristics of individual patients is required. In our study, the cuproptosis pattern characterized by the immune-inflamed phenotype and immune-desert phenotype showed lower and higher CRG_scores, respectively. Significant differences between the two cohorts in clinical prognostic features, gene mutations, immune infiltration, stromal score, MSI, Dysfunction, Exclusion, TIDE, TMB, and drug susceptibility were noted. Finally, we established a nomogram by integrating the patient’s age, tumor stage, and CRG_scores to improve the prediction model. This supports the accurate prognosis stratification of TNBC patients and a better understanding of the molecular mechanism of this disease, providing new ideas for tumor immunotherapy.

In breast cancer, elevated copper ions are associated with tumor progression, remodeling of the TME, and drug resistance (38). Currently, immune checkpoint inhibitors are the most widely used immunotherapy drugs for breast cancer in clinical research. The IMpassion 130 trial opened a new chapter in breast cancer immunotherapy and used PD-L1 as a mature biomarker for treatment of metastatic TNBC. However, because the screening population was limited to PD-1 positive patients, the results were narrow (39). Although we have achieved some success in using immunotherapy, the prognosis of TNBC patients is still significantly poor. Identifying more appropriate biomarkers for accurate treatment plan development is urgently needed (40). This highlights the crucial role of the TME in breast cancer tumorigenesis. TNBC has a unique TME that is related to cell proliferation, apoptosis, angiogenesis, immunosuppression, and drug resistance (41). Studies have shown that a considerable number of delta T cells in TNBC tumors can kill cancer cells through their innate immune functions. The high levels of CD4 memory resting and delta T cells are both significantly associated with OS (42, 43). This is consistent with our study, where CRG_score score was negatively correlated with the number of gamma delta T cells, CD4 memory resting T cells, and eosinophils in immune cells, suggesting that those with low CRG_scores have good prognoses. In addition, the levels of regulatory T cells, memory B cells, and plasma cells were significantly reduced in the low CRG_score group. Immunosuppression and immunodeficiency are two major functional characteristics of regulatory T cells. These cells are reprogrammed to proliferate and differentiate, which in turn changes the transcription profiles of infiltrating immune cells and support immune evasion. Moreover, a high abundance of regulatory T cells is significantly associated with high tumor expression levels of immune checkpoint inhibitor genes (44, 45), which may explain the effective response of the low CRG_score group to immunotherapy. Although the presence of B cells and plasma cells is a good predictor of patient prognosis (46, 47), B cells also have immunosuppressive roles in TNBC by enhancing the levels of myeloid-derived suppressor cells (MDSCs) or promoting IL-10 levels to facilitate isotype conversion to immunosuppressive IgG4 antibodies (48, 49). In fact, the effects of the humoral immune response on tumors may not be related to the presence of B cells (50), but rather depend on the tumor antigen recognition ability and activation of the complement signaling pathway, which ultimately determines the immune phenotype of B cells.

Tumors often have heterogeneous gene expression patterns and both the mRNA and protein levels. In recent years, significant heterogeneity has also been observed in the TME and its role in tumor development has been more appreciated (51). In addition, the inconsistency between research observations and clinical results of PD-1/PD-L1 inhibitors guided by biomarker expression levels has indicated that tumor immunotherapy is quite complex.

Microsatellites are widely distributed repetitive DNA motifs (52). The occurrence of new microsatellite alleles from the insertion or deletion of repeat units is known as MSI. In theory, H-MSI is often associated with a good prognosis. However, the incidence of dMMR/MSI-H in TNBC is actually extremely low and is not an appropriate representative independent prognostic factor for this disease (53). Studies have shown that Dysfunction and Exclusion can accurately predict the immunotherapy effect of tumors, and TIDE is a more optimal algorithm to simulate tumor immune escape. In our study, CRG_score was negatively correlated with TIDE, Dysfunction, and Exclusion, and positively correlated with MSI.

The P53 gene encodes a 43.7 KDa protein that is mainly distributed in the cytoplasm of cells. It can specifically bind to DNA, and its activity is regulated by post-translational modifications such as phosphorylation, acetylation, methylation, and ubiquitination. P53 is a tumor suppressor gene with extensive and powerful functions. More than half of tumors carry a p53 mutation, and its involvement in inhibiting innate immunity and immune escape have been demonstrated (54). P53 expression is not only related to the number of tumor-infiltrating lymphocytes and chemotherapy efficacy in TNBC patients (55, 56), but also promotes tumorigenesis by inducing the systemic circulation of EGFR (57).

Previously observed mutations of the TTN gene are mostly related to cardiomyopathy and skeletal muscle diseases. Recent studies have found that TTN is one of the most commonly mutated genes in various cancers, including metastatic TNBC. The mutation load of TTN can represent the high TMB status of the tumor, which has predictive value for immunotherapy response and prognosis (58, 59). The most significant TNBC somatic mutations in our study are P53 and TTN, which are consistent with the results of Lips (60), where these mutated genes are associated with relapse and chemotherapy resistance and may express better responses to immunotherapy.

Similarly, low CRG_score tumors also show H-TMB, a widely recognized biomarker for predicting the therapeutic effects of immune checkpoint blockade (ICB) (61). In summary, we conclude that the CRG_score, as a comprehensive biomarker integrating various other markers such as the TMB, TME status, and MSI status, is likely to be a more effective prediction strategy for immunotherapy response.

Our study has confirmed that the CRG_score can be used to comprehensively assess the CRG expression pattern and the corresponding TME immune cell infiltration characteristics in individual patients. This will help determine the immunophenotype of the tumor and guide a more accurate and effective clinical treatment plan. In addition, the CRG_score can also predict the efficacy of immunotherapy in TNBC patients as an independent prognostic biomarker. Our research results provide new ideas and directions for improving the clinical effects of immunotherapy in patients, identifying different TNBC immunophenotypes, and promoting accurate and personalized immunotherapy in the future.

## Conclusion

Our comprehensive analysis of CRGs has revealed the extensive regulatory effects of these genes on the TME, clinical features, and prognosis. In addition, we have determined the value of these CRGs in TNBC immunotherapy. These findings confirm the clinical significance of CRGs and provide new research directions for personalized and precise immunotherapy approaches.

## Data availability statement

Data are available in a public, open access repository. Data are available upon reasonable request. All data relevant to the study are included in the article or uploaded as supplementary information.

## Author contributions

ML,TW, HM and WL designed this study and directed the research group in all aspects, including planning, execution, and analysis of the study. SS, LS and XW drafted the manuscript. XW, SS, and YC collected the data. XW, LS, and HX provided the statistical software, performed the data analysis, SS,HX and YX arranged the Figures and Tables. SS, LS,XW and YX revised the manuscript. All authors contributed to the article and approved the submitted version.

## Funding

This study was supported in part by the Beijing Hongdingxiang Public Welfare Development Center (BJ-HDX-20211163).

## Conflict of interest

The authors declare that the research was conducted in the absence of any commercial or financial relationships that could be construed as a potential conflict of interest.

## Publisher’s note

All claims expressed in this article are solely those of the authors and do not necessarily represent those of their affiliated organizations, or those of the publisher, the editors and the reviewers. Any product that may be evaluated in this article, or claim that may be made by its manufacturer, is not guaranteed or endorsed by the publisher.
