# Supplementary figures and images for: Prognostic analysis of cuproptosis-related gene in triple-negative breast cancer
Source: Front Immunol. 2022 Aug 1;13:922780. doi: 10.3389/fimmu.2022.922780 (PMC9376234; doi:10.3389/fimmu.2022.922780)

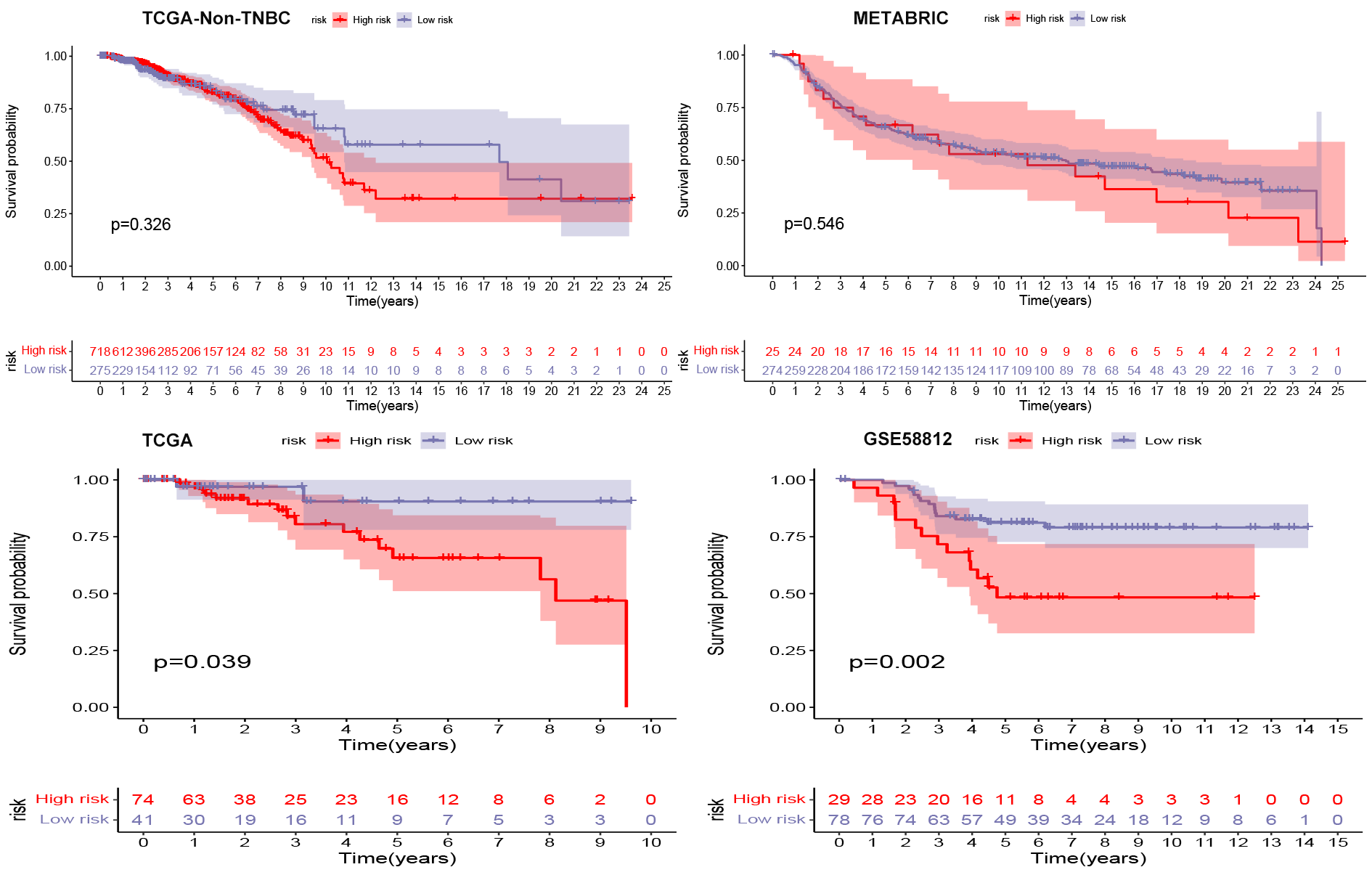

Supplement: Supplementary file 1 [file Image_1.tif]

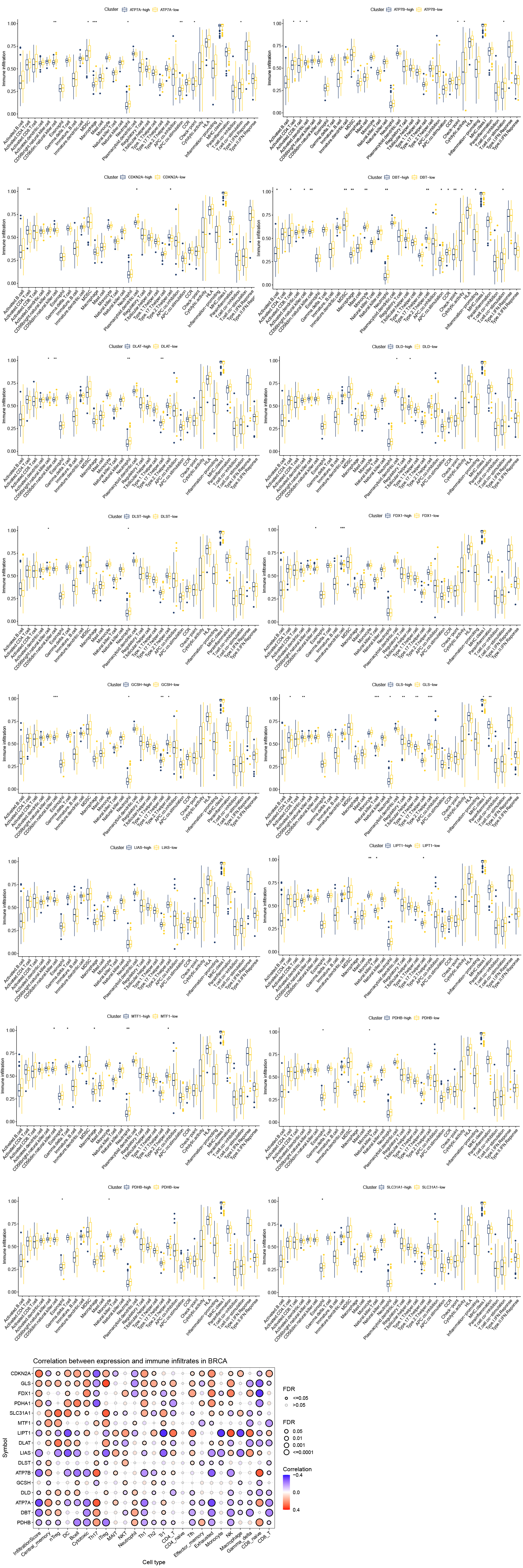

Supplement: Supplementary file 2 [file Image_2.tif]

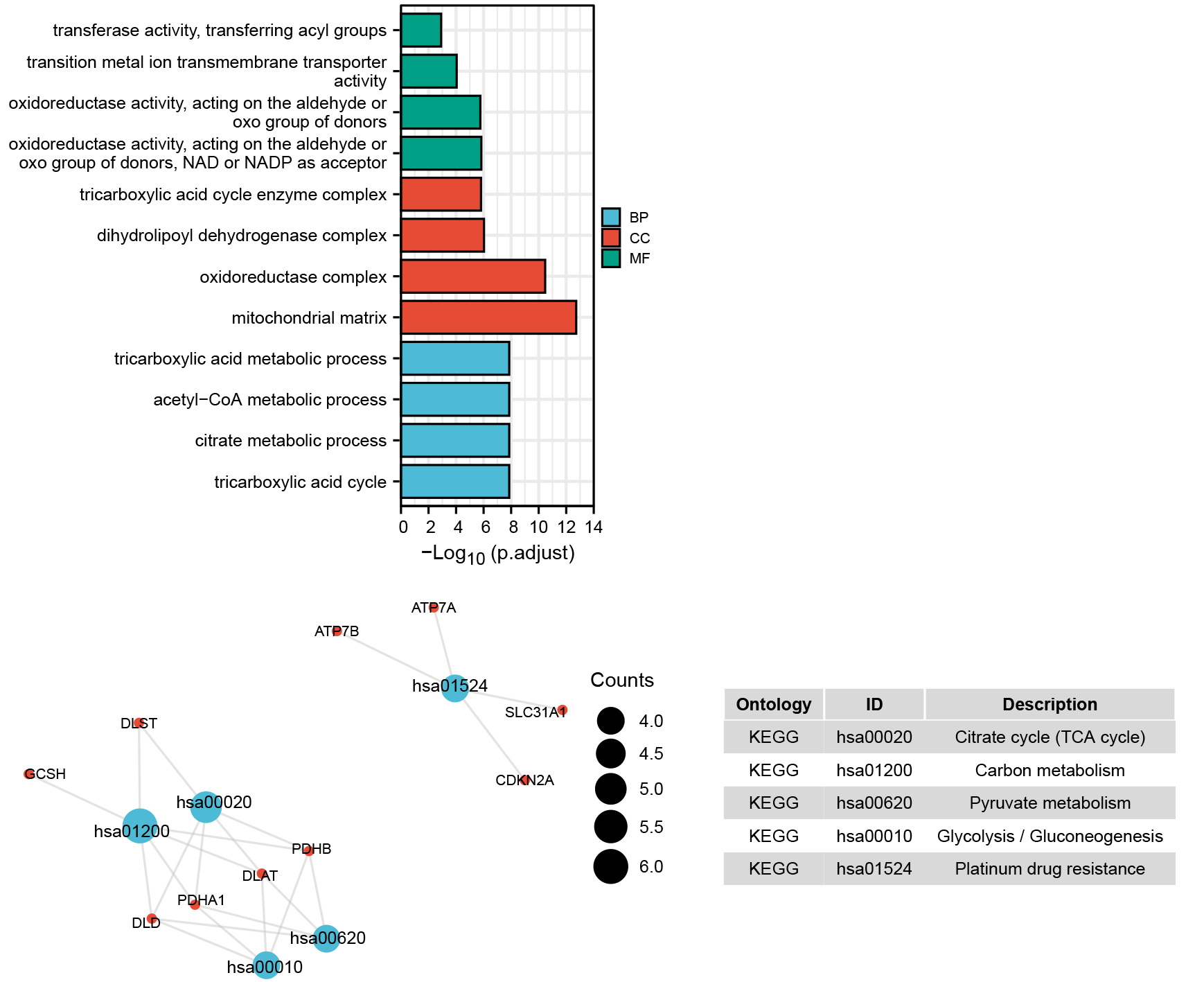

Supplement: Supplementary file 3 [file Image_3.tif]

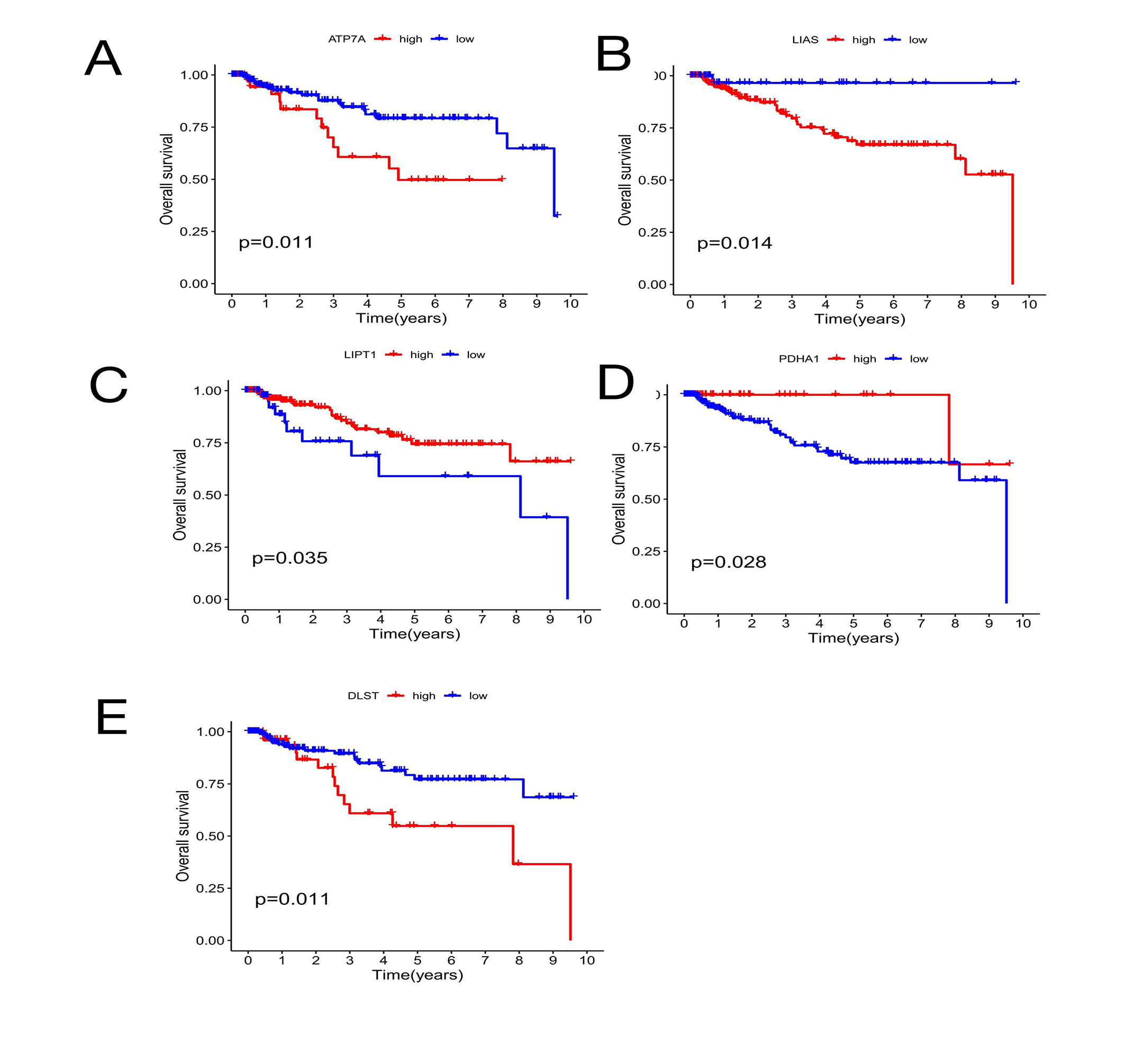

Supplement: Supplementary file 4 [file Image_4.tif]
